# Aggressive conflict tactics in romantic relationships: Examining the roles of trust and loneliness in a Cypriot sample

**DOI:** 10.1371/journal.pone.0357368

**Published:** 2026-09-23

**Authors:** Marilena Mousoulidou, Andri Christodoulou, Anna Maria Palli

**Affiliations:** Department of Psychology, Neapolis University Pafos, Paphos, Cyprus; Amity University Kolkata, INDIA

## Abstract

**Purpose:**

Aggressive conflict tactics in romantic relationships are an increasing concern, highlighting the importance of examining the emotional factors that may be associated with their use in the general population. This study aimed to assess the prevalence of aggressive conflict tactics among Greek-speaking adults in Cyprus and to explore their associations with demographic characteristics, relationship trust, and loneliness.

**Methods:**

In this cross-sectional design, 300 Greek-speaking adults residing in Cyprus (63% female; M age = 32.85 years) were recruited through convenience and snowball sampling. Participants completed an online questionnaire comprising the Revised Conflict Tactics Scale, the Trust in Close Interpersonal Relationships Scale, and the UCLA Loneliness Scale to assess aggressive conflict tactics, levels of trust in interpersonal relationships, and loneliness.

**Results:**

Findings suggest that (a) aggressive conflict tactics are highly prevalent, with psychological aggression reported by over 85% of participants and nearly 30% reporting physical assault; (b) sexual coercion was the only aggressive conflict tactic consistently associated with demographic variables, including gender, age, and education; (c) lower levels of trust were associated with the perpetration and victimization of physical assault, as well as victimization of sexual coercion and injury; and (d) higher loneliness was associated with sexual coercion perpetration.

**Conclusions:**

These findings highlight the importance of emotional factors in understanding the dynamics of aggressive conflict in romantic relationships. Trust and loneliness were associated with the use and experience of aggression, highlighting the need for relationship education programs that promote emotional safety, trust-building, and awareness around consent and communication.

## Introduction

Interpersonal relationships are a fundamental part of human well-being, playing a key role in emotional regulation, mental health, and identity. Changes in the nature or quality of these relationships are inevitable and may sometimes lead to interpersonal conflict. Conflict is deeply intertwined with cultural and psychological factors that influence its emergence and management [1]. Interpersonal conflict arises when individuals perceive their needs, goals, or values as incompatible, often resulting in tension and emotional strain [2]. Conflict may be managed through constructive or aggressive tactics, with the latter potentially undermining the functioning of the relationship. Disparities in values, threats to identity, and communication failures can intensify such conflict [2]. Because humans are inherently social beings, perceived or actual disconnection from meaningful relationships can trigger psychological distress comparable to physical pain [3]. These findings highlight the psychological complexity of interpersonal conflict and emphasize the importance of understanding the emotional and relational factors that influence how conflict is managed. These issues are particularly relevant in Greek-speaking populations, where relatively little research has examined the psychological factors associated with aggressive conflict tactics, especially among adults in Cyprus.

Conflict resolution tactics refer to the behavioral strategies individuals use to respond to and manage interpersonal conflict, particularly within close relationships [4,5]. These strategies are commonly categorized as constructive or destructive. Constructive tactics, such as open communication, negotiation, and mutual problem-solving, aim to preserve the relationship and resolve the issue collaboratively [5,6]. In contrast, destructive tactics, also referred to as aggressive tactics, include behaviors like withdrawal, verbal hostility, domination, and avoidance, which tend to escalate conflict and undermine relational stability [7,8]. Scholars have further classified conflict behaviors into categories that include constructive negotiation tactics (such as reasoning, compromise, emotional expression), as well as more harmful behaviors such as psychological aggression (verbal and non-verbal acts intended to cause emotional harm), physical assault (use or threat of physical force), sexual coercion (unwanted sexual activity), and injury (physical outcomes resulting from conflict) [4]. Conflict behaviors can also be understood in terms of motivational lines, with communal strategies driven by concern for a partner’s needs and agentic strategies reflecting self-interest and control [9]. These patterns are shaped not only by the immediate context of disagreement but also by earlier relational experiences. For example, experiencing family conflict or aggression during childhood has been linked to a higher chance of using aggressive tactics in romantic relationships later in life [8,10]. Overall, conflict tactics reflect both situational responses and long-term interpersonal patterns influenced by learned behaviors and attachment styles.

Existing literature on romantic relationships has shown that the use of aggressive conflict tactics, such as psychological aggression, physical violence, sexual coercion, and injury, is consistently linked to adverse relational outcomes. These include lower relationship satisfaction, emotional disengagement, and an increased risk of abuse and long-term relational damage [8,11]. In contrast, constructive tactics like emotional expression, compromise, and open negotiation are associated with greater relationship stability, higher satisfaction, and more effective emotional regulation [7]. These patterns highlight the importance of how partners choose to handle conflict and the significant effect these strategies can have on both the relationship and personal well-being.

Although aggressive conflict tactics represent one way of responding to interpersonal conflict, repeated or severe use of these behaviors may occur within the broader context of intimate partner violence (IPV) [12,13]. IPV is a pervasive and significant public health concern that includes physical, sexual, and psychological harm inflicted by a current or former partner or spouse. It includes various forms of abuse such as physical assault, sexual coercion, psychological aggression, stalking, financial abuse, and controlling behaviors, all of which function as mechanisms of power and domination [12–14]. According to national and international surveys, IPV affects a significant portion of the population across genders, with nearly 37.3% of women and 30.9% of men in the United States reporting experiences of sexual violence, physical violence, or stalking by an intimate partner [15]. Among young adults in Greece, high rates of psychological aggression and controlling behavior have been documented, suggesting that such patterns may be widespread across different cultural contexts [16]. The prevalence is particularly high among young adults, individuals with disabilities, and marginalized groups, highlighting the intersection between IPV and broader social inequalities [15]. Importantly, IPV can persist even after the end of a relationship and is associated with escalating patterns of control and violence, including threats, strangulation, and severe physical injuries [12,13]. Longitudinal findings further suggest that early relational conflicts may escalate into IPV if left unaddressed [17].

The psychological impact of IPV is severe, with survivors often suffering from depression, post-traumatic stress disorder (PTSD), anxiety, substance use disorders, suicidal thoughts, and other mental health conditions [13,14]. These consequences are often intensified by experiences of emotional humiliation, entrapment, and loss of autonomy and control. Moreover, destructive conflict, including withdrawal, verbal aggression, and psychological manipulation, may function not only as signs of dysfunctional communication but also as mechanisms of abuse within violent relationships [11,18]. For instance, repeated verbal aggression has been associated with coercive control and emotional disengagement in long-term romantic relationships [11]. Moreover, among Greek university students, high levels of psychological aggression have been reported, with females reporting higher use of negotiation but also of psychological tactics, indicating complex gender dynamics in conflict behavior [16]. Insecure attachment styles, particularly anxious attachment, have also been linked to the use and perception of destructive conflict resolution strategies, which increase the risk of psychological abuse and sexual coercion [18,19]. Similar associations have recently been reported among university students, where gender role expectations and attachment styles were related to psychological violence in intimate relationships [20]. Therefore, IPV involves not only physical safety but also underlying patterns of power imbalance, conflict avoidance, and emotional vulnerability.

One of the most significant factors associated with conflict resolution is trust. Trust has been conceptualized as a prospective expectation that others will act reliably and appropriately, integrating both cognitive evaluations and behavioral commitments [21]. In romantic relationships, trust represents the confident expectation that one’s partner will be reliable, caring, and attentive to the relationship’s needs [22,23], thereby allowing individuals to remain vulnerable to their partner’s intentions [19]. Trust is developed over time through repeated interactions in which the partner acts consistently and supportively [24]. According to attachment theory, trust originates from early caregiving experiences and manifests as a stable internal representation in adulthood. Conversely, interdependence theory conceptualizes trust as a dynamic and context-sensitive construct that emerges from relationship-specific interactions [19,25]. Trust has also been shown to facilitate emotional safety and is positively associated with the use of constructive conflict resolution tactics, thereby reinforcing relational stability [26].

Empirical research highlights the protective role of trust in romantic conflict. Individuals exhibiting higher levels of trust are more likely to use constructive conflict tactics, such as negotiation and forgiveness, and less likely to engage in aggressive behaviors, including contempt, emotional withdrawal, or psychological aggression [7,11,12]. Trust promotes benevolent interpretations of partner transgressions, thereby enhancing forgiveness and emotional closeness [7,23]. In contrast, individuals with low trust tend to perceive partner offenses as more severe, respond with less forgiveness, and exhibit heightened behavioral reactivity and control [12,27]. Even one low-trust partner can undermine the benefits of constructive strategies, leading to decreased post-conflict closeness and impaired communication [7]. Additionally, insecure attachment is strongly associated with lower dyadic trust and a greater reliance on harmful coping mechanisms when relational expectations are violated [19,22]. Higher trust is consistently associated with stronger relational bonds and relationship satisfaction [7,28], highlighting it not only as an outcome of relational functioning but also as a key factor in how couples engage in and recover from conflict.

Loneliness is another important emotional variable that is associated with relationship quality and conflict behavior. It is described as a subjective experience arising from a perceived gap between one's social connections and one's desires [29]. Unlike social isolation, loneliness is a psychological phenomenon that may persist even in the presence of others, especially when emotional or relational needs remain unmet [3]. According to the cognitive discrepancy model, loneliness arises when one’s current relationship fails to meet internal standards for emotional closeness [30]. Attachment theory further suggests that individuals with anxious or avoidant attachment styles are particularly susceptible to loneliness, as they often struggle to develop secure and emotionally responsive bonds [19,31]. Research in both adolescence and adulthood has linked loneliness to lower self-esteem, greater emotional withdrawal, and diminished relationship awareness [32].

Loneliness is linked to maladaptive relationship functioning, particularly in terms of trust and conflict behaviors. Research indicates that individuals who feel lonely are more likely to report lower trust in their romantic partners and to use unhealthy conflict resolution strategies, including psychological aggression, emotional disengagement, and avoidance [12,29]. Loneliness is negatively associated with relationship awareness, which co-occurs with heightened conflict and impaired emotional connection [27]. Low dyadic trust and limited partner responsiveness are associated with greater loneliness and reduced relationship satisfaction [22]. Similarly, unpredictable partner behavior, often experienced as emotional unreliability, is linked to feelings of disconnection and loneliness [24]. These findings suggest that loneliness is closely associated with reduced intimacy, unmet emotional needs, and aggressive conflict patterns.

Trust and loneliness are conceptually distinct but closely related constructs, and growing research suggests that they are interrelated in how individuals navigate conflict in romantic relationships. Loneliness is associated with lower relationship awareness and attentiveness, which are in turn associated with lower interpersonal trust and greater frequency and intensity of conflict [27,33]. When individuals feel disconnected, they are more likely to interpret partner behaviors more negatively and perceive lower emotional responsiveness, both of which relate to lower trust [3,24]. Conversely, low trust has been associated with increased social withdrawal, rumination, and emotional distancing, which reinforce feelings of loneliness [22,34].

Research suggests a reciprocal relationship between loneliness and trust, whereby loneliness may undermine trust and lower trust may reinforce feelings of loneliness, with both being associated with maladaptive conflict tactics, such as emotional reactivity, psychological aggression, and avoidance [12,29]. Partners with low trust are more likely to display contempt and less likely to forgive during conflict, both of which relate to lower post-conflict closeness and relational satisfaction [7,35]. These patterns may become more pronounced when one or both partners exhibit high psychological inflexibility, which has been associated with greater difficulty regulating negative affect and maintaining emotional presence during relational distress [27]. In relationships where trust and intimacy are low, conflict resolution may shift from cooperative negotiation to adversarial control, particularly among individuals with attachment-related vulnerabilities or past trauma [31,34]. Together, these findings suggest that loneliness and trust are closely interrelated and jointly associated with how romantic partners engage in, experience, and recover from conflict.

The current study focuses on examining aggressive conflict tactics among Greek-speaking adults in Cyprus. Even though previous findings from Greece underscore the urgency of addressing aggressive conflict tactics in adult relationships [16], recent reports indicate that interpersonal violence also remains a significant public health concern in Cyprus [36–38]. Among a sample of 247 university students, over 80% acknowledged psychological aggression, approximately one-third encountered physical assault, over a quarter reported perpetrating sexual coercion, and 40.6% reported being victims of sexual coercion. Men reported higher rates of perpetration in severe forms of aggressive conflict tactics [16]. These findings highlight the pervasive nature of relational aggression within Greek-speaking populations and underscore the importance of examining the emotional and interpersonal factors associated with these patterns.

The alarming rates of interpersonal violence reported in Cyprus in recent years further highlight the importance of this issue. According to the Cyprus Association for the Prevention and Handling of Domestic Violence (SPAVO) [36,37], 318 women and 437 children sought refuge services in 2023 alone, with nearly all adult clients reporting psychological abuse, and a majority also disclosing physical (59%), economic (32%), or sexual violence (18%). Police statistics in Cyprus further illustrate the escalation of domestic violence over time, with reported cases rising from 965 in 2018 to over 3000 annually in subsequent years. Victims were predominantly women (66.2%), but men and minors were also impacted [38]. Significantly, these figures likely underestimate the true extent of the problem, as stigma, emotional dependency, and institutional mistrust often deter victims from reporting. These findings from both Greece and Cyprus underscore the need to enhance our understanding of the emotional and relational factors that contribute to aggressive conflict behaviors. While local data highlight the prevalence and severity of relational aggression, especially among young adults within families, they also reveal a gap in addressing the psychological processes that are associated with such dynamics. This context highlights the importance of examining psychological and relational dynamics, especially trust, loneliness, and conflict resolution tactics, that may be associated with violence in intimate relationships.

Despite growing interest in the individual roles of trust, loneliness, and conflict in romantic relationships, empirical studies that examine these constructs in an integrated framework remain limited. Most existing research has focused on the independent associations of either trust or loneliness on relational outcomes [7,22,29], overlooking the combined associations of these factors on conflict behaviors. For example, studies on trust often emphasize its role in forgiveness and reduced aggression [7], while loneliness research tends to highlight its association with emotional dysregulation and lower relationship satisfaction [32]. However, these lines of inquiry rarely intersect. To date, few studies have examined how trust and loneliness jointly relate to the use of constructive or aggressive conflict resolution tactics [27,33], particularly in Greek-speaking populations.

The current study aims to investigate the relationship between trust and loneliness and the use of aggressive conflict tactics in romantic relationships among the general adult population of Cyprus. To the best of our knowledge, no prior study in Cyprus has examined trust, loneliness, and aggressive conflict tactics within a single integrated framework. Most existing studies have either focused solely on emotional predictors, such as trust or loneliness [7,22,29], or have explored conflict tactics without considering how emotional vulnerabilities may be associated with their use [16]. Although recent data reveal alarming rates of domestic and relational violence in Cyprus [36–38], little is known about the psychological factors associated with these behaviors in the general population. Thus, the current study addresses these gaps by examining trust and loneliness simultaneously in relation to aggressive conflict tactics among adults in Cyprus while also considering relevant demographic characteristics. Based on these objectives, the following hypotheses were formulated:

**Hypothesis 1 (H1).** *The prevalence of aggressive conflict is expected to be comparable to previous findings in Greek-speaking samples, which reported frequent use of psychological, physical, and sexual aggression in romantic relationships.*

**Hypothesis 2 (H2).** *There will be associations between demographic characteristics and aggressive conflict tactics. Consistent with prior research, gender differences are expected, while age- and education-related patterns will be examined in an exploratory manner.*

**Hypothesis 3 (H3).** *There will be negative associations between trust and aggressive conflict tactics. In line with the existing literature, higher levels of trust are expected to be associated with lower reports of both perpetrating and experiencing aggressive tactics.*

**Hypothesis 4 (H4).** *There will be positive associations between loneliness and the use of aggressive conflict tactics. Based on existing literature, higher levels of loneliness are expected to be related to higher reports of perpetrating and experiencing aggressive tactics.*

## Materials and methods

### Sample

The study’s final sample included 300 individuals, of whom 189 were female (63%). The sample's age ranged from 18 to 67 years, with a mean age of 32.85. Among the participants, 98 (32.7%) were emerging adults (18–25 years), 119 (39.7%) were in early adulthood (26–39 years), and 83 (27.7%) were in middle-to-late adulthood (40 + years). Regarding education level, 96 participants (32%) had a lower education (secondary schooling), 124 participants (41.3%) had a middle education (a bachelor’s degree), and 80 participants (26.7%) had a higher education (a master’s or doctoral degree). All participants were Greek speakers residing in Cyprus.

### Measures

*Demographic Information:* Data were collected on participants’ age, gender, and education level.

*Relationship Tactics:* To assess conflict resolution tactics in close relationships, the Revised Conflict Tactics Scale (CTS2) [4] ([16] for the Greek version) was employed. The CTS2 is a 78-item questionnaire that asks participants to report how often each behavior occurred within the past year, using a frequency-based response format: (1) once in the past year, (2) twice in the past year, (3) 3–5 times in the past year, (4) 6–10 times in the past year, (5) 11–20 times in the past year, (6) more than 20 times in the past year, (7) not in the past year, but it did happen before, (8) this has never happened. The questionnaire is divided into five subscales: four capturing aggressive tactics: (a) psychological aggression with 16 items (e.g., I shouted or yelled at my partner), (b) physical assault with 24 items (e.g., I threw something at my partner that could hurt), (c) sexual coercion with 14 items (e.g., I insisted on sex when my partner did not want to – but did not use physical force), and (d) injury with 12 items (e.g., I had a sprain, bruise, or small cut because of a fight with my partner), and one capturing constructive tactics: (e) negotiation with 12 items (e.g., I showed my partner I cared even though we disagreed). Each aggressive tactic subscale includes items reflecting minor or severe acts, while the negotiation subscale captures both emotional and cognitive strategies. All 78 items are presented in pairs, 39 items that reflect the respondent’s behavior and 39 items that reflect their partner’s behavior. For aggressive tactics, this distinction allows assessment of perpetration and victimization, while for negotiation, it reflects self-use and partner-use. The consistency reliability for the CTS2 subscales in the current sample ranged from low to very good, with Cronbach’s alpha values as follows: psychological aggression (α = 0.645 perpetration, α = 0.773 victimization), physical assault (α = 0.703 perpetration, α = 0.853 victimization), sexual coercion (α = 0.660 perpetration, α = 0.507 victimization), injury (α = 0.683 perpetration, α = 0.703 victimization), and negotiation (α = 0.811 self-use, α = 0.780 partner-use).

*Relationship Trust:* To measure participants’ levels of trust in their relationship partner, the Trust in Close Interpersonal Relationship Scale [39]. As no validated Greek version of the scale was available, the instrument was translated into Greek following a standard forward-backward translation procedure. The process included independent forward translation, back-translation, and expert review to ensure conceptual and linguistic equivalence. The scale includes 18 items that measure the level of trust in close interpersonal relationships (e.g., I know how my partner is going to act. My partner can always be counted on to act as I expect), rated on a 7-point Likert-type scale ranging from 1 (strongly disagree) to 7 (strongly agree). The Greek version of the scale measures a single total trust score, with higher scores indicating greater levels of trust. The coefficient alpha for the current sample was excellent, with a Cronbach’s alpha value of 0.911.

*Loneliness:* To assess participants’ level of loneliness, the UCLA Loneliness Scale [40] ([41] for the Greek version) was employed. The scale includes 20 items (e.g., How often do you feel there are people you can turn to?), which are rated on a 4-point Likert-type scale ranging from 1 (never) to 4 (often). Total scores range between 20 and 80, with higher scores indicating a greater level of loneliness. In the present study, we used both the total score of loneliness as well as the classification scheme used by Deckx [42]: (a) low degree of loneliness (score between 20 and 34), (b) moderate degree of loneliness (score between 35 and 49), (c) moderately high degree of loneliness (score between 50 and 64) and high degree of loneliness (score between 65 and 80). The scale demonstrated excellent internal consistency for the current sample, with a Cronbach’s alpha of 0.919.

### Procedure

After approval by the Cyprus National Bioethics Committee (ref no: EEBK/EΠ/2024/86, 6 February 2025), the study was conducted in accordance with the principles of the Declaration of Helsinki. Written informed consent was obtained electronically from all participants prior to participation. The questionnaires used in the current study were compiled into a single questionnaire created via Google Forms. Data were collected via convenience and snowball sampling methods between 11 March 2025 and 30 May 2025 to recruit as many participants as possible. Participation was restricted to Greek-speaking adults aged 18 years or older residing in Cyprus who were currently in a romantic relationship of at least one year’s duration. This eligibility criterion was stated in both the recruitment advertisement and the participant information sheet presented before participants accessed the questionnaire in Google Forms. Data collection continued until a sample of 300 participants had been obtained. The sample size was not based on a priori power analysis but rather on practical feasibility within the planned data collection period and was comparable to previous regional research on aggressive conflict tactics in romantic relationships [16]. Participant recruitment involved advertisements on social networks (Facebook and Instagram), social chat applications (Viber and WhatsApp), and private emails sent to friends, colleagues, organized groups, and students. Prior to participation, all participants were informed about the study’s purpose, duration, and their rights, including the guarantee of anonymity and the voluntary nature of participation. Informed consent to participate was obtained electronically by pressing a consent checkbox before accessing the questionnaire. Completion time was approximately 15 minutes.

### Data processing and measures

After data collection, preliminary data processing and scoring were conducted. For demographic information, age and education categories were grouped into meaningful categories. Age was categorized based on developmental psychology age classifications [43]: (a) the emerging adulthood group, which included participants aged between 18 and 25, (b) the early-adulthood group, which included participants aged between 26 and 39, and (c) the middle-to-late adulthood group, which included participants aged 40 years and older. For education, three categories were created based on participants’ education level: (a) the lower education group, which included participants who completed secondary education, (b) the middle education group, which included participants who completed their bachelor’s, and (c) the higher education group, which included participants who completed their master’s or doctoral degree. Consistent with previous CTS2 research [16], participants’ responses were processed to derive indices of past-year prevalence and chronicity. Following the CTS2 scoring guidelines [44], responses indicating a behavior had occurred during the previous year were converted into frequency scores, with response options 1–6 assigned values of 1, 2, 4, 8, 15, and 25, respectively. These values were used to estimate the chronicity (i.e., annual frequency) of each aggressive tactic among participants reporting at least one incident during the previous year. The primary analytic approach involved creating binary variables, indicating whether each type of behavior had occurred at least once within the past year (1 = present, 0 = absent). This approach was selected because the primary objective was to examine past-year prevalence of each aggressive conflict tactic and to permit consistent binary logistic regression analyses across all CTS2 subscales. However, dichotomization reduces information regarding the frequency and variability of aggressive acts; therefore, frequency-based chronicity estimates were retained for the descriptive analyses. This binary classification was applied across the four aggressive subscales (i.e., psychological aggression, physical assault, sexual coercion, and injury), as well as the negotiation subscale. For each aggressive subscale, binary scores were calculated for minor, severe, and combined acts, with the combined category indicating whether participants reported at least one minor or severe act during the past year. For negotiation, binary scores were computed separately for emotional, cognitive, and combined strategies, based on self-use and partner-use items. Responses referring only to events that occurred before the past year were excluded from the prevalence and chronicity analyses but were summarized descriptively in a separate analysis. Consequently, prevalence and chronicity were calculated separately for each CTS2 subscale. Because each subscale was scored independently, participants contributed only to analyses for which a past-year classification could be established, resulting in slight differences in sample size across analyses. There were no missing questionnaire responses. The varying analytic sample sizes therefore did not result from listwise or pairwise deletion, but from independent scoring of each CTS2 subscale according to the past-year scoring procedure. For trust, the Trust in Close Relationships Scale was used to calculate the total trust score for each participant. Finally, the total score of the UCLA Loneliness Scale was used to categorize participants into four levels of loneliness: (a) low loneliness, (b) moderate loneliness, (c) moderately high loneliness, and (d) high loneliness.

### Statistical analysis

All data were entered into the Statistical Package for Social Sciences, Version 29.0 (SPSS 29, IBM Corporation, Armonk, NY, USA); the significance level for all tests was set at 5%; and all the necessary analyses were performed accordingly. No listwise or pairwise deletion was required because there were no missing questionnaire responses. Analytic sample sizes varied only because CTS2 subscales were scored independently as described above. The binary logistic regression analyses served as the primary multivariable analyses, whereas the descriptive, chi-square, and independent-samples t-test analyses were used to characterize the data and examine bivariate associations. Given the number of bivariate analyses conducted, no formal correction for multiple comparisons was applied; therefore, these findings were interpreted cautiously and considered complementary to the multivariable regression models. Preliminary analyses included calculating descriptive statistics for the various demographic factors. Descriptive statistics were also computed to examine the prevalence, severity, and chronicity of each aggressive tactic as measured by the CTS2, separately for perpetration and victimization. This included frequencies and means of incidents reported within the past year, as well as reports of aggression that occurred exclusively before the past year. Additionally, the prevalence of negotiation strategies was calculated based on the presence of emotional and cognitive tactics. To examine gender, age, and education differences in aggressive tactics, chi-square tests of independence were conducted, separately for perpetration and victimization. Next, independent-samples t-tests were used to assess differences in trust total scores between individuals who reported perpetrating or experiencing each aggressive tactic and those who did not. For loneliness, independent-samples t-tests were conducted to compare loneliness total scores between participants who reported and those who did not report each of the eight aggressive tactics. Chi-square analyses were also used to explore how loneliness categories (i.e., low, moderate, moderately high) varied by the tactic. For the binary logistic regression analyses, categorical predictors were entered as categorical variables, with female, age 18–25 years, and lower education as the reference categories. Finally, binary logistic regression analyses were performed to examine whether trust, loneliness, gender, age, and education were associated with reporting each aggressive tactic. Multicollinearity diagnostics indicated no evidence of problematic collinearity among the predictors (all VIFs ≤ 1.07).

## Results

To examine Hypothesis 1, descriptive analyses were conducted to assess the prevalence, severity, and chronicity of aggressive conflict tactics reported solely within the past year. As shown in Table 1, psychological aggression was the most prevalent form of aggression for both perpetration and victimization. Psychological aggression also demonstrated the highest chronicity. Physical assault was the second most prevalent aggressive tactic, with approximately one-third of participants reporting perpetration or victimization, and around half of these cases involving severe acts. Sexual coercion was less prevalent than psychological aggression and physical assault, but was associated with relatively high chronicity among participants who reported these behaviors. Finally, injury was the least prevalent aggressive tactic, although participants who reported injury experienced multiple incidents over the previous year. These findings support Hypothesis 1.

**Table 1:** Prevalence and chronicity of reported perpetration and victimization of aggressive conflict tactics during the past year.

				Prevalence			Chronicity		
Type of Aggressive Tactic (CTS2)					95% CI^a^			95% CI^b^	
	Severity	N	n	P	LL	UL	M	LL	UL
**Perpetrating Aggression Against Partner**									
Psychological Aggression	Severe	268	69	25.8	20.9	31.3	7.3	5.2	9.3
	Combined	280	240	85.7	81.1	89.3	13.6	11.6	15.6
Physical Assault	Severe	282	39	13.8	10.3	18.4	9.4	5.5	13.3
	Combined	270	76	28.2	23.1	33.8	10.8	6.9	14.7
Sexual Coercion	Severe	299	25	8.4	5.7	12.1	7.0	3.7	10.3
	Combined	285	57	20.0	15.8	25.0	11.5	6.9	16.0
Injury	Severe	298	21	7.1	4.7	10.5	9.1	4.2	14.0
	Combined	292	37	12.7	9.3	17.0	8.2	4.8	11.7
**Victim of Aggression by Partner**									
Psychological Aggression	Severe	262	72	27.5	22.4	33.2	8.2	6.0	10.5
	Combined	280	233	83.2	78.4	87.1	15.0	12.5	17.6
Physical Assault	Severe	282	35	12.4	9.1	16.8	8.5	4.1	12.9
	Combined	268	79	29.5	24.3	35.2	9.7	5.6	13.7
Sexual Coercion	Severe	288	25	8.7	6.0	12.5	6.7	3.5	10.4
	Combined	273	69	25.3	20.5	30.8	11.0	7.5	14.5
Injury	Severe	292	20	6.9	4.5	10.3	7.2	5.1	9.2
	Combined	283	45	15.9	12.1	20.6	5.8	4.1	7.5

**Note.** N = number of participants included in the analysis for the corresponding CTS2 subscale; n = number of participants reporting the corresponding aggressive tactic during the past year; P = prevalence (%); M = mean frequency (chronicity); CI^a^ = 95% CI for prevalence calculated using the Wilson’s Score Method; CI^b^ = 95% CI for mean frequency based on standard error; LL = lower limit; UL = upper limit. Sample sizes vary across subscales because each CTS2 subscale was scored independently using the CTS2 past-year scoring procedure.

Furthermore, conflict tactics that occurred before the past year were also examined. As shown in Table 2, a smaller proportion of participants reported perpetrating or experiencing aggression exclusively before the past year. Physical assault was the most frequently reported tactic, followed by psychological aggression, sexual coercion, and injury. These findings indicate that a small proportion of participants reported aggressive conflict tactics exclusively before the previous year.

**Table 2:** Descriptive statistics of aggressive conflict tactics reported before the past year.

Aggressive Tactic Type (CTS2)	Perpetration n (%)	Victimization n (%)
Psychological Aggression	20 (6.7%)	20 (6.7%)
Physical Assault	30 (10%)	32 (10.7%)
Sexual Coercion	15 (5%)	27 (9%)
Injury	8 (2.7%)	17 (5.7%)

**Note.** Frequencies represent participants who reported perpetrating or experiencing at least one minor or severe tactic before, but not during, the past year. Percentages calculated using the total sample (N = 300).

As part of the preliminary analyses, it was also considered essential to examine the constructive aspects of participants’ relationship dynamics. Table 3 depicts the participants’ use of emotional and cognitive negotiation strategies during conflict. Negotiation strategies were reported by nearly all participants for both self-use and partner-use, with emotional strategies reported slightly more frequently than cognitive strategies. Since nearly all participants reported using negotiation tactics, resulting in highly uneven group sizes, this variable was excluded from subsequent analyses.

**Table 3:** Descriptive statistics of constructive tactics reported by participants.

Constructive Tactic Type (CTS2)	Self-Use n (%)	Partner-Use n (%)
Emotional Negotiation	288 (96%)	287 (95.7%)
Cognitive Negotiation	287 (95.7%)	285 (95%)
Any	289 (96.3%)	289 (96.3%)

**Note.** Percentages calculated out of the total sample (N = 300).

To examine Hypothesis 2 and possible gender differences in the presence or absence of each aggressive conflict tactic, chi-square tests of independence were conducted. Most chi-square analyses did not reveal significant associations between gender and perpetrating or experiencing aggression. Specifically, no statistically significant relationships were found for psychological aggression perpetration, *χ*²(1, N = 280) = 0.04, *p* = .839, *φ* = .012; psychological aggression victimization, *χ*²(1, N = 280) = 0.42, *p* = .515, *φ* = .039; physical assault perpetration, *χ*²(1, N = 270) = 0.53, *p* = .467, *φ* = .044; physical assault victimization, *χ*²(1, N = 268) = 0.001, *p* = .975, *φ* = .002; sexual coercion victimization, *χ*²(1, N = 273) = 0.25, *p* = .618, *φ* = .030; injury perpetration, *χ*²(1, N = 292) = 0.28, *p* = .599, *φ* = .031; and injury victimization, *χ*²(1, N = 283) = 1.36, *p* = .243, *φ* = .069. The only significant association emerged for sexual coercion perpetration, *χ*²(1, N = 285) = 13.07, *p* < .001, *φ* = .214. A higher proportion of men than women reported sexual coercion perpetration during the past year (31.1% vs. 13.4%). Overall, the findings suggest that only sexual coercion perpetration differed significantly by gender.

To further explore demographic influences on aggression, chi-square tests of independence were conducted to examine whether perpetration or victimization of aggressive conflict tactics differed across age groups (i.e., emerging adulthood, early adulthood, and middle-to-late adulthood). Most analyses did not reveal statistically significant associations between age group and aggressive tactics: psychological aggression perpetration, χ²(2, N = 280) = 1.13, *p* = .569, Cramer’s *V* = .063; psychological aggression victimization, χ²(2, N = 280) = 0.04, *p* = .981, Cramer’s *V* = .012; physical assault perpetration, χ²(2, N = 270) = 5.19, *p* = .075, Cramer’s *V* = .139; physical assault victimization, χ²(2, N = 268) = 0.18, *p* = .915, Cramer’s *V* = .026; sexual coercion perpetration, χ²(2, N = 285) = 4.64, *p* = .098, Cramer’s *V* = .128; injury perpetration, χ²(2, N = 292) = 0.01, *p* = .995, Cramer’s *V* = .006; and injury victimization, χ²(2, N = 283) = 1.22, p = .544, Cramer’s *V* = .066. Although the overall Pearson chi-square tests for physical assault perpetration and sexual coercion perpetration were not statistically significant, the corresponding linear-by-linear associations were significant (*p* = .027 and *p* = .040, respectively). The only statistically significant association was found for sexual coercion victimization, χ²(2, N = 273) = 10.65, *p* = .005, Cramer’s *V* = .197. A significantly higher percentage of individuals in emerging adulthood reported experiencing sexual coercion in the past year (37.4%) compared to those in the early adulthood age group (20.0%) and the middle-to-late adulthood group (18.1%). Overall, these findings suggest that age differences were primarily found for sexual coercion victimization.

Then, chi-square tests of independence were conducted to examine whether perpetration or victimization of aggressive conflict tactics differed across education groups (i.e., lower education, middle education, and higher education). Most analyses indicated no statistically significant relationship between education level and perpetrating or experiencing aggressive tactics. Chi-square tests did not yield statistically significant results for psychological aggression perpetration, χ²(2, N = 280) = 1.73, *p* = .420, Cramer’s *V* = .079; psychological aggression victimization, χ²(2, N = 280) = 0.50, *p* = .779, Cramer’s *V* = .042; physical assault perpetration, χ²(2, N = 270) = 1.37, *p* = .505, Cramer’s *V* = .071; physical assault victimization, χ²(2, N = 268) = 2.71, *p* = .258, Cramer’s *V* = .101; sexual coercion victimization, χ²(2, N = 273) = 4.25, *p* = .120, Cramer’s *V* = .125; injury perpetration, χ²(2, N = 292) = 2.40, *p* = .301, Cramer’s *V* = .091; and injury victimization, χ²(2, N = 283) = 2.69, *p* = .261, Cramer’s *V* = .097. The only statistically significant finding emerged for sexual coercion perpetration, χ²(2, N = 285) = 7.34, *p* = .026, Cramer’s *V* = .160. Participants in the middle education group were most likely to report perpetration (24.6%), followed by those with lower education (22.8%), while those with higher education reported the lowest prevalence (9.3%). Overall, these findings provide partial support for Hypothesis 2, with statistically significant demographic differences observed only for sexual coercion.

To examine Hypothesis 3, independent-samples t-tests were computed to assess statistically significant differences between aggressive conflict tactics (i.e., psychological aggression, physical assault, sexual coercion, and injury) and trust total score, analyzed separately for perpetration and victimization. These analyses were based on the presence of any minor or severe incident reported within the last year. As shown in Table 4, participants who reported either perpetrating or experiencing aggression generally reported lower levels of trust toward their partners compared to those who did not. Statistically significant differences were observed for all tactics across both perpetration and victimization, with moderate to large effect sizes. The only exception was the tactic of sexual coercion perpetration, which did not reach statistical significance. Overall, the findings largely support Hypothesis 3, indicating that lower levels of trust are associated with greater involvement in both the perpetration and experience of aggressive tactics.

**Table 4:** Independent-samples t-tests comparing trust scores based on reported aggressive conflict tactics in the past year.

Perpetrating Aggression Against Partner							
Aggressive Tactic Type (CTS2)	n Never Used	n Used Past Year	M(SD) Never Used	M(SD) Used Past Year	t(df)	p	Cohen’s d
Psychological Aggression	40	240	99.2 (22.9)	89.2 (20.6)	2.8 (278)	0.005**	0.48
Physical Assault	194	76	93.3 (21.4)	80.9 (18.6)	4.4 (268)	<0.001**	0.60
Sexual Coercion	228	57	91.0 (21.4)	89.3 (20.2)	0.6 (283)	0.573	0.08
Injury	255	37	91.7 (20.8)	83.1 (20.9)	2.36 (290)	0.019*	0.42
**Victim of Aggression by Partner**							
Psychological Aggression	47	233	98.8 (22.9)	88.8 (20.4)	3.0 (278)	0.003**	0.48
Physical Assault	189	79	95.4 (19.1)	79.2 (20.8)	6.2 (266)	<0.001**	0.83
Sexual Coercion	204	69	94.0 (19.8)	82.8 (22.0)	4.0 (271)	<0.001**	0.55
Injury	238	45	93.1 (19.7)	79.2 (23.1)	4.2 (281)	<0.001**	0.68

**Note.** * = *p* < .05; ** = *p* < .01; Aggressive tactic type = participants who endorsed at least one minor or severe tactic in the past year. Sample sizes vary across aggressive conflict tactics because each CTS2 subscale was scored independently using the CTS2 past-year scoring procedures.

Before examining Hypothesis 4, the distribution of participants’ loneliness levels was analyzed. Table 5 presents the number and percentage of participants falling into each category based on their total UCLA loneliness scale scores. As shown, only nine participants were categorized as experiencing “High Loneliness.” Because this group was too small to permit reliable comparisons, these participants remained in the descriptive analyses but were excluded from analyses involving loneliness categories. However, they were retained in analyses using the continuous loneliness total score (i.e., independent-samples t-tests and binary logistic regression analyses).

**Table 5:** Distribution of loneliness levels in the current sample.

	Low	Moderate	Moderately High	High
N	79	144	68	9
Percent	26.3%	48%	22.7%	3%

**Note.** Categories are based on total scores from the UCLA Loneliness Scale; percentages are calculated from the full sample (N = 300).

To examine Hypothesis 4, independent-samples t-tests were conducted to investigate statistically significant differences between the aggressive conflict tactics (i.e., psychological aggression, physical assault, sexual coercion, and injury) and loneliness total score, separately for perpetration and victimization. Table 6 presents the results. Participants who reported perpetrating or experiencing aggressive tactics in the past year generally reported higher levels of loneliness compared to those who did not. Significant differences were observed for all tactics in both perpetration and victimization, except injury perpetration, with small to moderate effect sizes.

**Table 6:** Independent-samples t-tests comparing loneliness total scores based on reported aggressive conflict tactics in the past year.

Perpetrating Aggression Against Partner							
Aggressive Tactic Type (CTS2)	n Never Used	n Used Past Year	M(SD) Never Used	M(SD) Used Past Year	t(df)	p	Cohen’s d
Psychological Aggression	40	240	38.0 (8.6)	43.0 (11.1)	−2.7 (278)	0.007**	−0.46
Physical Assault	194	76	41.1 (11.5)	45.7 (8.8)	−3.5† (177.27)	<0.001**	−0.43
Sexual Coercion	228	57	41.4 (11.1)	45.5 (9.8)	−2.6 (283)	0.011*	−0.38
Injury	255	37	41.6 (11.0)	45.0 (10.3)	−1.8 (290)	0.076	−0.31
**Victim of Aggression by Partner**							
Psychological Aggression	47	233	38.7 (9.0)	43.1 (11.2)	−2.5 (278)	0.013*	−0.40
Physical Assault	189	79	40.9 (11.2)	45.7 (9.5)	−3.6† (170.13)	<0.001**	−0.45
Sexual Coercion	204	69	40.4 (10.5)	46.4 (11.3)	−4.1 (271)	<0.001**	−0.57
Injury	238	45	41.0 (10.6)	46.8 (11.4)	−3.3 (281)	0.001**	−0.54

**Note.** * = *p* < .05; ** = *p* < .01; † = Levene’s test for equality of variances was significant, Welch’s t-test reported (equal variances not assumed); Aggressive tactic type = participants who endorsed at least one minor or severe tactic in the past year. Sample sizes vary across aggressive conflict tactics because each CTS2 subscale was scored independently using the CTS2 past-year scoring procedure.

To further examine the association between loneliness and aggressive conflict tactics, chi-square tests were computed for each aggressive tactic and loneliness level (i.e., low, moderate, and moderately high) for both perpetration and victimization. The chi-square test for psychological aggression perpetration showed a statistically significant relationship with loneliness type, *χ*^*2*^(2, N = 271) = 10.8, *p* = .005, Cramer’s *V* = .200. For the non-perpetrators, the majority fell into the moderate loneliness category (65%), followed by low (32.5%) and moderately high (2.5%) categories. Among perpetrators, although the majority also fell into the moderate loneliness category (48.5%), a larger proportion reported moderately high loneliness (26%) and a slightly smaller proportion reported low loneliness (25.5%). The chi-square test for psychological aggression victimization was also statistically significant, *χ*^*2*^(2, N = 271) = 8.8, *p* = .012, Cramer’s *V* = .180. Among non-victims, the majority reported moderate loneliness (61.7%), followed by low (31.9%) and moderately high (6.4%) levels. In contrast, among victims, although most also reported moderate loneliness (48.2%), the proportion reporting moderately high loneliness was higher than among non-victims (26.3%), and the proportion reporting low loneliness was lower (25.4%). The chi-square test for physical assault perpetration showed a statistically significant relationship with loneliness type, *χ*^*2*^(2, N = 261) = 18.2, *p* < .001, Cramer’s *V* = .264. Among non-perpetrators, the majority reported moderate loneliness (46.8%), followed by low (33.9%) and moderately high (19.4%). Among perpetrators, while the majority also reported moderate loneliness (56.0%), a greater proportion reported moderately high loneliness (34.7%), and a lower proportion reported low loneliness (9.3%). The chi-square test for physical assault victimization also showed a statistically significant association with loneliness type, *χ*^*2*^(2, N = 260) = 15.1, *p* < .001, Cramer’s *V* = .241. Among non-victims, the majority reported moderate loneliness (46.4%), followed by low (33.3%) and moderately high (20.2%) levels. In contrast, among victims, although the majority fell into the moderate loneliness category (58.4%), the proportion reporting moderately high loneliness was higher (31.2%), and the proportion reporting low loneliness was lower (10.4%). The chi-square test for sexual coercion perpetration showed a statistically significant relationship with loneliness type, *χ*^*2*^(2, N = 276) = 9.7, *p* = .008, Cramer’s *V* = .188. Among non-perpetrators, the majority reported moderate loneliness (49.8%), followed by low (30.8%) and moderately high (19.5%). Among perpetrators, while the majority also reported moderate loneliness (49.1%), the proportion reporting moderately high loneliness was higher (36.4%), and the percentage reporting low loneliness was lower (14.5%). The chi-square test for sexual coercion victimization showed a statistically significant relationship with loneliness type, *χ*^*2*^(2, N = 264) = 13.2, *p* = .001, Cramer’s *V* = .223. Among non-victims, the majority reported moderate loneliness (50.3%), followed by low (32.7%) and moderately high (17.1%). Among victims, most participants also reported moderate loneliness (46.2%), however, the proportion reporting moderately high loneliness was higher (36.9%), while the percentage reporting low loneliness was lower (16.9%) than that of non-victims. The chi-square test for injury perpetration did not show a statistically significant association with loneliness type, *χ*^*2*^(2, N = 283) = 4.0, *p* = .138, Cramer’s *V* = .118. Among participants reporting injury perpetration, a higher proportion reported moderately high loneliness (33.3%) compared to non-perpetrators (21.1%). The chi-square test for injury victimization showed a statistically significant relationship with loneliness type, *χ*^*2*^(2, N = 274) = 7.1, *p* = .029, Cramer’s *V* = .160. Among non-victims, the majority reported moderate loneliness (50.2%), followed by low (30.5%) and moderately high (19.3%). In contrast, among victims, although the majority fell into the moderate loneliness category (46.3%), the proportion reporting moderately high loneliness was higher (36.6%), and the proportion reporting low loneliness was lower (17.1%). Overall, the findings largely support Hypothesis 4, indicating that higher levels of loneliness are associated with greater involvement in both the perpetration and victimization of aggressive tactics.

A series of eight binary logistic regression analyses was conducted to evaluate potential associations between five independent variables (i.e., trust total score, loneliness total score, gender, age, and education) and the likelihood of individuals reporting either the perpetration or victimization of the four aggressive conflict tactics. Prior to interpretation, model fit was assessed using the Hosmer-Lemeshow goodness-of-fit test. None of the logistic regression models showed evidence of poor fit (all Hosmer-Lemeshow ps > .05), indicating adequate model fit. Overall, five of the eight models were statistically significant (see Table 7). The model for physical assault perpetration correctly classified 70.4% of the cases. Similarly, the model for physical assault victimization achieved a classification accuracy of 75%. The model for sexual coercion perpetration correctly classified 80% of the cases, while the model predicting sexual coercion victimization demonstrated a classification accuracy of 76.2%. Lastly, the model for injury victimization correctly classified 85.2% of the cases. In contrast, three models did not reach statistical significance. Specifically, the models predicting psychological aggression perpetration, psychological aggression victimization, and injury perpetration did not achieve significance. Within these three non-significant models, none of the five predictors (i.e., trust total score, loneliness total score, gender, age, and education) reached statistical significance.

**Table 7:** Binary logistic regression models for perpetration and victimization of aggressive conflict tactics.

Aggressive Tactic Type (CTS2)	Model χ^2^ (df = 7)	R^2^	Predictor	B	p	OR (95% CI)
**Perpetrating Aggression Against Partner**						
Psychological Aggression	12.529	.078	Trust	−0.015	.144	0.985 (0.966–1.005)
			Loneliness	0.032	.103	1.033 (0.993–1.074)
			Male (vs. Female)	0.127	.740	1.135 (0.536–2.403)
			Age 26–39 (vs. 18–25)	−0.047	.915	0.954 (0.399–2.281)
			Age ≥ 40 (vs. 18–25)	−0.329	.483	0.719 (0.287–1.806)
			Education Middle (vs. Lower)	−0.506	.257	0.603 (0.251–1.447)
			Education Higher (vs. Lower)	−0.243	.633	0.784 (0.289–2.125)
Physical Assault	23.862	.122	Trust	**−0.024**	**.002**	**0.976** **(0.961–0.991)**
			Loneliness	0.014	.374	1.014 (0.984–1.045)
			Male (vs. Female)	−0.131	.665	0.877 (0.485–1.587)
			Age 26–39 (vs. 18–25)	−0.375	.259	0.687 (0.358–1.318)
			Age ≥ 40 (vs. 18–25)	−0.654	.086	0.52 (0.246–1.098)
			Education Middle (vs. Lower)	−0.02	.951	0.98 (0.509–1.887)
			Education Higher (vs. Lower)	−0.13	.739	0.878 (0.409–1.885)
Sexual Coercion	32.884	.172	Trust	0.01	.278	1.01 (0.992–1.028)
			Loneliness	**0.049**	**.006**	**1.05** **(1.014–1.087)**
			Male (vs. Female)	**1.371**	**<.001**	**3.94** **(2.024–7.669)**
			Age 26–39 (vs. 18–25)	−0.521	.155	0.594 (0.290–1.217)
			Age ≥ 40 (vs. 18–25)	**−0.955**	**.026**	**0.385** **(0.166–0.891)**
			Education Middle (vs. Lower)	0.128	.720	1.136 (0.565–2.283)
			Education Higher (vs. Lower)	−0.7	.160	0.497 (0.187–1.318)
Injury	8.371	.053	Trust	−0.017	.087	0.983 (0.964–1.002)
			Loneliness	0.01	.612	1.01 (0.972–1.049)
			Male (vs. Female)	0.192	.609	1.212 (0.580–2.535)
			Age 26–39 (vs. 18–25)	0.193	.655	1.213 (0.520–2.830)
			Age ≥ 40 (vs. 18–25)	0.141	.768	1.151 (0.452–2.932)
			Education Middle (vs. Lower)	0.098	.81	1.103 (0.497–2.447)
			Education Higher (vs. Lower)	−0.585	.278	0.557 (0.193–1.604)
**Victim of Aggression by Partner**						
Psychological Aggression	11.470	.067	Trust	−0.019	.051	0.981 (0.963–1.000)
			Loneliness	0.023	.220	1.023 (0.987–1.060)
			Male (vs. Female)	0.258	.476	1.294 (0.636–2.634)
			Age 26–39 (vs. 18–25)	0.149	.707	1.161 (0.533–2.529)
			Age ≥ 40 (vs. 18–25)	0.166	.708	1.18 (0.497–2.803)
			Education Middle (vs. Lower)	−0.088	.827	0.916 (0.416–2.017)
			Education Higher (vs. Lower)	−0.044	.924	0.957 (0.389–2.354)
Physical Assault	36.281	.180	Trust	**−0.039**	**<.001**	**0.962** **(0.946–0.978)**
			Loneliness	0.005	.768	1.005 (0.974–1.036)
			Male (vs. Female)	−0.018	.952	0.982 (0.542–1.781)
			Age 26–39 (vs. 18–25)	0.111	.747	1.117 (0.569–2.193)
			Age ≥ 40 (vs. 18–25)	0.268	.486	1.308 (0.615–2.779)
			Education Middle (vs. Lower)	−0.044	.895	0.957 (0.495–1.849)
			Education Higher (vs. Lower)	−0.377	.346	0.686 (0.313–1.502)
Sexual Coercion	30.985	.158	Trust	**−0.018**	**.027**	**0.982** **(0.966–0.998)**
			Loneliness	0.031	.050	1.032 (1.000–1.065)
			Male (vs. Female)	0.414	.190	1.512 (0.815–2.807)
			Age 26–39 (vs. 18–25)	**−0.759**	**.027**	**0.468** **(0.239–0.916)**
			Age ≥ 40 (vs. 18–25)	**−0.899**	**.025**	**0.407** **(0.186–0.892)**
			Education Middle (vs. Lower)	0.195	.568	1.215 (0.623–2.371)
			Education Higher (vs. Lower)	−0.219	.606	0.803 (0.349–1.848)
Injury Victimization	23.834	.138	Trust	−0.029	**.003**	**0.972** **(0.954–0.990)**
			Loneliness	0.023	.210	1.023 (0.987–1.061)
			Male (vs. Female)	0.513	.149	1.671 (0.832–3.355)
			Age 26–39 (vs. 18–25)	−0.131	.756	0.878 (0.385–1.999)
			Age ≥ 40 (vs. 18–25)	0.33	.452	1.391 (0.589–3.288)
			Education Middle (vs. Lower)	0.317	.425	1.374 (0.630–2.995)
			Education Higher (vs. Lower)	−0.203	.688	0.817 (0.304–2.192)

**Note.** B = regression coefficient; OR = odds ratio; CI = confidence interval; R² = Nagelkerke pseudo-R². The reference categories for the categorical predictors were female (gender), 18–25 years (age), and lower (education). Trust and loneliness were entered as continuous variables. Model χ² values are omnibus likelihood ratio tests. Statistically significant predictors (*p* < .05) are shown in **bold**.

As shown in Table 7, trust emerged as the sole statistically significant predictor for physical assault perpetration, where higher levels of trust were associated with a lower likelihood of reporting perpetration. Similarly, higher trust scores were associated with reduced odds of physical assault victimization. For sexual coercion perpetration, three variables emerged as significant predictors: loneliness, age group, and gender. Specifically, higher levels of loneliness were related to increased odds of perpetration; participants in the middle-to-late adulthood group (40 years and older) were less likely to report perpetration compared to those in the emerging adulthood group; and male participants exhibited higher odds of reporting sexual coercion perpetration relative to female participants. For sexual coercion victimization, significant associations were observed for trust and age group. Higher trust scores were associated with lower odds of victimization. Additionally, compared to the youngest reference age group (i.e., emerging adults), participants in early adulthood and middle-to-late adulthood demonstrated lower odds of experiencing victimization. Lastly, for injury victimization, higher trust scores corresponded to a lower likelihood of reporting victimization.

## Discussion

The current study adds new evidence to the literature on romantic relationship dynamics by examining the use of aggressive conflict tactics and their associations with trust and loneliness among adults in Cyprus. Responding to a growing concern about interpersonal violence in Cyprus, this study explored the associations of emotional and relational factors with the use of psychologically and physically harmful behaviors in romantic conflict. The findings revealed high rates of aggressive conflict tactics, with psychological aggression being the most prevalent form reported. While certain demographic disparities were observed, these were concentrated primarily in sexual coercion outcomes and were related to gender, age, and education, while no consistent patterns emerged across other forms of aggression or demographic categories. Bivariate analyses generally linked lower levels of trust and higher levels of loneliness with greater involvement in aggressive conflict tactics, whereas the adjusted regression models revealed more outcome-specific associations. Taken together, these results contribute to the existing literature by demonstrating the association among emotional and relational factors, such as trust and loneliness, and the use of aggressive conflict tactics in romantic relationships.

First, the current findings supported Hypothesis 1 by revealing a high prevalence of aggressive conflict tactics among adults in Cyprus, suggesting that psychologically and physically harmful behaviors were commonly reported in romantic relationships within this sample. Psychological aggression was the most common form, reported by most participants (over 85%), both as perpetrators and victims. A substantial portion of these incidents involved severe psychological tactics, highlighting the emotional intensity of partner interactions. Nearly 30% of the participants also reported physical assault, and over one in four participants disclosed either perpetrating or experiencing sexual coercion. Although injury was the least frequently reported form, it still affected a notable proportion of respondents. This pattern of results closely aligns with findings from a Greek-speaking student sample reported by Kalaitzaki et al. [16], where similarly high rates of psychological aggression and notable levels of physical and sexual aggression were documented. When considering official statistics from Cyprus, which report over 3,000 domestic violence incidents annually and widespread experiences of psychological, physical, and sexual abuse [36–38], the current findings emphasize the severity and widespread nature of aggression in intimate relationships. However, these findings should be interpreted cautiously because the CTS2 assesses a broad range of aggressive behaviors, including relatively minor acts of psychological aggression, such as shouting or insulting a partner. Consequently, the prevalence estimates reported here are not directly comparable with official domestic violence statistics, which primarily reflect incidents reported to authorities. Despite these considerations, the present findings suggest that aggressive conflict tactics are commonly reported within intimate relationships in the present sample. At the same time, nearly all participants reported using emotional and cognitive negotiation strategies, suggesting that constructive and aggressive conflict tactics may coexist within romantic relationships. Furthermore, only a relatively small proportion of participants reported using aggressive tactics exclusively before the past year, indicating that the high prevalence observed primarily reflected behaviors occurring during the study’s reference period.

Second, the current study findings provided partial support for Hypothesis 2. Demographic differences in the use of aggressive conflict tactics were limited. While previous research has often emphasized the role of gender in shaping conflict behaviors (e.g., [16]), our findings revealed that most forms of aggression did not significantly differ between men and women. The only exception was sexual coercion perpetration, where men were more than twice as likely to report engaging in it (31.1% vs. 13.4%). This pattern was also reflected in the multivariate analyses, where male participants had nearly four times the odds of reporting sexual coercion perpetration after adjustment for trust, loneliness, age, and education. Additionally, sexual coercion was the only aggressive tactic for which demographic differences consistently emerged across the analyses. This pattern may suggest that developmental and social factors are particularly relevant to sexual coercion. Research has shown that normative beliefs about relationships and sex, such as the idea that persistence in sexual pursuit is acceptable or that consent is negotiable, are internalized by both men and women and may normalize coercive behaviors in intimate contexts [45,46]. Social norms surrounding sexuality, including traditional sexual scripts that emphasize male initiation and female gatekeeping, may further obscure the boundaries of consent, especially among younger individuals with limited relationship experience [45,47]. Additionally, feelings of entitlement and control, which are shaped by broader gendered power structures, may reinforce coercive behavior among men, especially when peer or cultural environments do not explicitly challenge these behaviors [48]. Together, these findings highlight the complex interplay of cultural norms, developmental influences, and perceived power dynamics that may explain why sexual coercion, unlike other forms of aggression, showed consistent demographic variation in our sample. They also highlight the importance of targeted educational and prevention efforts that promote consent awareness, challenge normative beliefs about gender and power, and foster healthy relational development, particularly among younger individuals.

Third, the findings largely supported Hypothesis 3 by identifying trust as an important psychological factor associated with aggressive conflict tactics. Lower levels of relationship trust were associated with greater involvement in most forms of aggressive conflict tactics, both as perpetrators and victims, including psychological aggression, physical assault, sexual coercion victimization, and injury. These associations were observed in the group comparisons and, after accounting for loneliness and demographic characteristics, trust remained independently associated with lower odds of physical assault perpetration and victimization, sexual coercion victimization, and injury victimization. These findings align with existing literature suggesting that low trust in romantic relationships is associated with greater emotional insecurity, negative attributions, and maladaptive responses to perceived threats during conflict [7,12,23]. The current findings extend previous research by suggesting that diminished trust is associated with greater involvement in several forms of aggressive conflict tactics. In emotionally intimate contexts where vulnerability is high, low trust may be associated with more intense conflict, poorer emotion regulation, and greater likelihood of harmful tactics. Conversely, higher trust may represent a potentially protective relational factor associated with a lower likelihood of certain aggressive conflict tactics. People who have more confidence in their partner’s reliability and emotional responsiveness may be better at handling conflict in a positive way and less likely to act destructively. These findings suggest that interventions promoting emotional safety, predictability, and trust within romantic relationships may help support healthier conflict resolution. Interestingly, trust did not significantly predict sexual coercion perpetration, highlighting once again the distinctive nature of this tactic. This supports our earlier finding that sexual coercion was the only aggressive tactic for which demographic differences consistently emerged across the analyses, whereas emotional factors such as trust showed more limited associations. These patterns may indicate that sexual coercion may be associated with different psychological and social processes, possibly involving normative beliefs, entitlement, or power dynamics more than interpersonal trust. This highlights the importance of tailored prevention strategies that go beyond individual emotional vulnerabilities.

Finally, the findings largely supported Hypothesis 4 by demonstrating an association between loneliness and the use of aggressive conflict tactics. Higher loneliness scores were significantly associated with greater involvement in perpetrating and experiencing psychological aggression, physical assault, and sexual coercion, as well as injury victimization. These associations were observed in the group comparisons and, after accounting for trust and demographic characteristics, loneliness remained independently associated with higher odds of sexual coercion perpetration. This pattern suggests that some of the observed associations between loneliness and aggressive conflict tactics may be explained by shared variance with trust or demographic characteristics. Participants reporting higher levels of loneliness, reflecting greater emotional disconnection and unmet relational needs, were more likely to engage in or experience aggression within their romantic relationships. These findings align with previous research suggesting loneliness is associated with poorer relationship functioning, including reduced emotional attunement and greater conflict reactivity [27,29]. One possible interpretation is that loneliness may be accompanied by reduced sensitivity to relational cues, more hostile interpretations of partner behavior, and less constructive communication during conflicts. The present results extend previous research by suggesting that loneliness is associated with greater involvement in several forms of aggressive conflict tactics. Unlike trust, which may help prevent conflict from escalating, loneliness may exacerbate relational vulnerabilities, possibly by heightening feelings of frustration, emotional neglect, or insecurity. These insights suggest that addressing loneliness may be beneficial as part of conflict prevention and relationship education. Enhancing emotional connectedness and increasing access to social and psychological resources may help decrease reliance on harmful conflict strategies and boost relationship resilience. Interventions that promote emotional availability, responsiveness, and secure attachment could be beneficial for individuals experiencing ongoing loneliness in romantic relationships.

The findings of this study are important given the widespread concerns about relationship aggression among adults. Overall, the findings suggest that relationship trust and loneliness are important psychological and relational factors associated with aggressive conflict tactics. From a theoretical perspective, these findings are broadly consistent with attachment theory, which proposes that insecurity within close relationships may undermine emotional regulation and increase maladaptive responses during interpersonal conflict [19]. They are also consistent with interdependence theory, which conceptualizes trust as a dynamic, relationship-specific process that develops through ongoing interpersonal interactions and shapes responses to relationship challenges [25]. Due to the high frequency of aggressive tactics, particularly psychological aggression, together with the observed associations between emotional factors and aggression, the study suggests the need for early intervention strategies that foster trust, emotional closeness, and healthy conflict resolution. These strategies could be included in relationship education, public health programs, or mental health services in various settings. Additionally, these findings support further research to explore how emotion regulation, attachment styles, and communication patterns contribute to understanding the relationship between trust, loneliness, and conflict behaviors. A better understanding of these processes may help inform more effective efforts aimed at reducing relationship aggression and strengthening romantic partnerships in different populations.

Although the current study provides valuable insights into the relationship between aggressive conflict tactics, trust, and loneliness in romantic relationships, several limitations must be acknowledged. Firstly, the study employed snowball and convenience sampling methods, which may not fully represent the broader population of Cyprus. Specific demographic subgroups, such as older adults, individuals from rural areas, or those with varying educational backgrounds, may have been inadequately represented, thereby limiting the generalizability of the findings. Furthermore, the cross-sectional design constrains the ability to establish causality. Secondly, the present study relied solely on self-report measures, which may have introduced social desirability and recall biases. As all constructs were assessed using the same response method, shared method variance may also have inflated some of the observed associations. In addition, although participants were required to meet the study’s relationship eligibility criteria, relationship-specific characteristics beyond these criteria were not systematically assessed. Variables such as relationship duration, cohabitation status, and relationship satisfaction were not accounted for in the present study, despite their potential influence on aggressive conflict tactics. Moreover, although the sample included adults across various age groups and educational backgrounds, there was a predominance of female participants, which may have limited the generalizability of some findings. Additionally, the sample size was determined by practical feasibility rather than an a priori power analysis. Furthermore, sexual orientation was not assessed and therefore could not be examined as a potential correlate of aggressive conflict tactics, limiting the generalizability of the findings across different relationship populations. Moreover, while the study emphasized trust and loneliness as key relational and emotional factors associated with aggression, other relevant psychological factors were not explored. Additionally, the internal consistency of the sexual coercion victimization subscale was relatively low (α = .507). Consequently, findings from this subscale should be interpreted with caution, as measurement error may have attenuated some of the observed associations. Lastly, although the statistical analyses were guided by the study hypotheses, the relatively large number of statistical tests may have increased the risk of Type I error. Therefore, the findings should be interpreted with appropriate caution until replicated in future studies.

Future research should consider longitudinal methodologies to better understand the directionality of the observed associations, including whether emotional vulnerabilities such as low trust and loneliness precede aggressive conflict tactics or whether repeated exposure to such conflicts contributes to diminished trust and heightened loneliness over time. Future studies should also conduct an a priori power analysis to ensure adequate statistical power, particularly for less prevalent outcomes and subgroup comparisons. Future investigations could incorporate qualitative methodologies, partner reports, or dyadic research designs to complement self-reported data and provide a more nuanced understanding of relational dynamics. Additionally, future research should aim for greater gender balance and investigate additional psychological factors, such as attachment styles, emotion regulation, and communication patterns, which may help explain why some individuals are more prone to emotional insecurity and conflict escalation than others. Future studies should also examine relationship-specific characteristics, such as relationship duration, cohabitation status, and relationship satisfaction, as these factors may influence aggressive conflict tactics. Examining these processes may provide a more comprehensive understanding of the pathways to relational aggression and inform the development of more targeted prevention and intervention strategies.

## Conclusions

In conclusion, this study provides valuable insights into the psychological and relational factors associated with aggressive conflict tactics in romantic relationships among adults in Cyprus. The findings revealed that aggressive tactics, particularly psychological aggression, were highly prevalent, with many individuals reporting both perpetrating and experiencing these behaviors. Moreover, sexual coercion was the only aggressive tactic consistently associated with demographic variables such as gender, age, and education, whereas other forms of aggression showed few demographic differences. The findings also showed that lower relationship trust and higher loneliness were generally associated with greater involvement in the perpetration and experience of aggressive conflict tactics. Although these associations became more selective after accounting for demographic characteristics and the overlap between trust and loneliness, the results highlight the importance of these emotional and relational factors in understanding aggressive conflict behaviors.

Overall, the findings emphasize the importance of strengthening emotional connections, building trust, and promoting healthy conflict resolution within romantic relationships. Public health programs, relationship education, and mental health services could benefit from including training on emotional responsiveness and healthy conflict resolution. This study adds to the existing literature by examining trust, loneliness, and aggressive conflict behaviors within a unified framework and provides evidence from a Greek-speaking adult population in Cyprus that may inform future research, policy, and practice aimed at promoting healthier romantic relationships.
